# Discovery of Novel MDR-*Mycobacterium tuberculosis* Inhibitor by New FRIGATE Computational Screen

**DOI:** 10.1371/journal.pone.0028428

**Published:** 2011-12-02

**Authors:** Christoph Scheich, Zoltán Szabadka, Beáta Vértessy, Vera Pütter, Vince Grolmusz, Markus Schade

**Affiliations:** 1 Combinature Biopharm AG, Berlin, Germany; 2 Department of Computer Science, Eötvös University, Budapest, Hungary; 3 Institute of Enzymology, Hungarian Academy of Science, Budapest, Hungary; 4 Uratim Ltd., Budapest, Hungary; 5 Department of Applied Biotechnology, University of Technology and Economics, Budapest, Hungary; Semmelweis University, Hungary

## Abstract

With 1.6 million casualties annually and 2 billion people being infected, tuberculosis is still one of the most pressing healthcare challenges. Here we report on the new computational docking algorithm FRIGATE which unites continuous local optimization techniques (conjugate gradient method) with an inherently discrete computational approach in forcefield computation, resulting in equal or better scoring accuracies than several benchmark docking programs. By utilizing FRIGATE for a virtual screen of the ZINC library against the *Mycobacterium tuberculosis* (*Mtb*) enzyme antigen 85C, we identified novel small molecule inhibitors of multiple drug-resistant *Mtb*, which bind in vitro to the catalytic site of antigen 85C.

## Introduction

All protein-ligand docking programs used for high throughput virtual screening use scoring functions for evaluating the relative positions of ligands and macromolecules [1], [2]. Mathematical optimization techniques are applied to find the best scoring position of the ligand in the macromolecule. With the additional need to allow ligand flexibility, this search for the best ligand position corresponds to a mathematical optimization problem of high dimensional space: the 3D position of the rigid small molecule can be described by 3 real numbers describing one atom of the small molecule plus 3 real numbers describing the Euler-angles. Every rotatable bond adds one additional dimension. Therefore a small molecule with 8 rotatable bonds needs to be optimized in the 3+3+8 = 14-dimensional real space. Computationally optimizing complex energy-like scoring functions of small molecule – macromolecule pairs in 14-dimensional space becomes a formidable task. In this work we compare our solution, the FRIGATE docking program, with 8 benchmark docking programs and demonstrate that FRIGATE yields promising small molecule ligands for the *Mycobacterium tuberculosis* (*Mtb*) enzyme antigen 85C.

Tuberculosis (TB) is the second highest cause of death from infectious disease, killing 1.6 million people annually. An estimated one third of the world population is latently infected with *Mtb*, the causative agent of TB [3]. While vaccination is largely ineffective in preventing adult pulmonary disease, the WHO recommended multi-drug treatment comprises 2 months of directly observed therapy with isoniazid, rifampicin, pyrazinamide and ethambutol followed by a minimum of 4 months of isoniazid and rifampicin. The complexity and duration of this treatment leads to poor disease control and the emergence of multi drug-resistant (MDR) and extensive drug-resistant (XDR) TB. Present second-line antibiotics for the treatment of resistant TB are inherently inadequate either due to limited efficacy or associated toxicities, indicating a high medical need for more field-effective anti-tuberculars [4].

The discovery of efficacious anti-tuberculars is particularly demanding due to the mycolic acid shield of the mycobacterial cell wall, which is essential for both viability and virulence of *Mtb* and targeted by the first-line anti-tuberculars isoniazid and ethambutol [5]. Cell wall mycolic acids are β-branched, γ-hydroxy fatty acids of 70 to 90 carbon atoms occurring as esters of arabinogalactan-peptidoglycan (mAGP) and trehalose, an α-1,1′-glucose disaccharide [6]. The transfer of mycolic acids from trehalose monomycolate (TMM) to trehalose dimycolate (TDM, cord factor) is catalyzed by the transferases antigen 85A, B and C (Ag85A, B, C) [7], which possess an almost invariant active site composed of a catalytic serine oxyanion positioned between the trehalose binding site and an extended hydrophobic channel thought to harbor the mycolic acid chain [8], [9].

Genetic knock-out of Ag85C in *Mtb* suppresses the amount of cell-wall linked mycolic acids by 40%, and knock-out of the Ag85A gene results in loss of *Mtb* replication in human and mouse macrophages [10]. RNA interference knock-down of Ag85A, B and C slows down *Mtb* growth in broth by 2 log units [11]. Covalent small molecule blockage with 6-azido-6′-deoxytrehalose weakly inhibits the growth of *Mycobacterium aurum* with a minimum inhibitory concentration (MIC) of 200 µg/mL [7]. Immunological analysis of TB infected patients shows that Ag85A, B, C and D belong to the major *Mtb* antigens, indicating that these proteins are accessible to immune cells and probably small molecules. Considering these data and the therapeutic success of other cell wall biosynthesis inhibitors, we chose Ag85C as a promising surrogate target for the discovery of new anti-tuberculars by computational screening.

Here we report the identification of the novel binder N-[3-(1H-imidazol-1-yl)propyl]-1-benzyl-9-methyl-4-oxo-1,4-dihydropyrido[1,2-*a*]pyrrolo[2,3-*d*]pyrimidine-2-carboxamide (**1**) to the catalytic site of Ag85C by the novel FRIGATE virtual screening algorithm. The binding site of **1** is experimentally confirmed by protein-detected NMR spectroscopy. Moreover we demonstrate that **1** inhibits the growth of *Msmeg* and MDR-*Mtb* in liquid broth at MICs of 50 and 20 µg/mL, respectively.

## Results and Discussion

### FRIGATE facilitates local optimization of ligand poses

Conceptually virtual screening is one of the fastest and most resource sparing approaches for identifying drug-like ligands to protein targets of known 3D structure. The scoring functions of virtual screening algorithms are frequently related to the molecular energies or potential functions. The exact description of the force fields in each geometric point around a target molecule is not possible, since there are infinitely many points. An approximation of the force field with a finite number of points, arranged in a cubic grid, is the usual solution, as embedded in the program AutoDock [12]. However, this discretization of the force field exacerbates the local optimization (energy minimization) because derivation and gradient methods need continuous, differentiable potential functions.

The new FRIGATE docking software of Uratim Ltd. [13] applies a novel hybrid approach: it discretizes the energy potentials around a protein molecule in order to be computationally feasible, and then uses a continuous local optimization technique, namely the scaled conjugate gradient (SCG) algorithm [14], for the twice continuously differentiable B-spline interpolation of the force field, based on discretized data points. The energy-based algorithm of FRIGATE computes the total free energy change, ΔG, from the sum of intermolecular and intramolecular terms as follows:
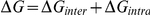



The intermolecular energy terms are given by the equation:
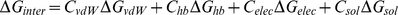
where on the right hand side of the equation sums the terms for van der Waals energy, hydrogen-bonding energy, electrostatic energy and solvation energy, respectively. Each term is weighted with a coefficient, determined from experimental binding constants using linear regression analysis, according to reference [12]. The intramolecular energy term, ΔG_intra_, describes the intramolecular van der Waals interactions and the torsional energy terms in the docked ligand.

### The FRIGATE program consists of the following parts

#### (a) Protein preparation

The three-dimensional structures of the proteins are taken from a PDB formatted file. The coordinates of the missing H atoms are computed, and partial charges are assigned to atoms, in order to compute later the electrostatic potential, the fragmental volume and the solvation energy terms. Directional parameters for computing hydrogen bonding energy terms for (i) oxygen atoms in the protein and (ii) H atoms in the protein are also calculated. The parameters gained are stored in the Receptor Specification File (RSF) of the protein.

#### (b) Grid calculation

Similarly to the AUTODOCK program [12], FRIGATE pre-computes the energy function around the protein molecule in the points of a three dimensional rectangular grid. Each possible ligand atom (i.e., polar hydrogen, aliphatic carbon, aromatic carbon, nitrogen, oxygen, phosphorus, sulfur, fluorine, chlorine, bromine, iodine, non-polar hydrogen) is placed in each possible grid point, and the energy affecting that atom is calculated and stored in a file called gridmap. The closest grid points are 0.375 Å apart which is roughly a quarter of a carbon-carbon bond length. Pre-computing and storing of the gridmap facilitates the fast computation of the energies of millions of flexible ligand conformations relative to the static protein molecule.

#### (c) Spline approximation

The pre-computed energy values of the discrete grid facilitate the fast computation of energy terms for ligand atoms that are *exactly positioned* in the grid points. Energies affecting ligand atoms *between* discrete grid points are computed from an approximation of the energies of the surrounding grid points. Unlike other programs, FRIGATE applies a twice continuously differentiable B-spline approximation function for this goal, therefore the energy function affecting the ligand atoms can be minimized locally with conjugate gradient methods [14], [17].

#### (d) Local minimization

The Scaled Conjugate Gradient (SCG) algorithm is applied for local minimization. It is detailed in the Methods section in Text S1.

#### (e) Global minimization

The Competitive MultiStart algorithm is used for global optimization. It is detailed in the Methods section in Text S1.

### Parameterization of FRIGATE

We optimized the SCG algorithm of FRIGATE against the thorough dataset of 100 high-resolution protein-ligand crystal structures utilized previously for benchmarking the eight popular docking programs DOCK, FLEXX, FRED, GLIDE, GOLD, SLIDE, SURFLEX and QXP [15]. These 100 public crystal structures comprises 97 diverse drug-like ligands, as evidenced by ligand molecular weights between 88 and 730 Da, total polar surface areas between 20 and 210 Å^2^ [16]. For our FRIGATE training dataset we generated 100 random ligand conformations for each of the 100 protein-ligand complexes. We observed that the SCG algorithm performs much slower when the resulting local optimum was in the positive region of the function. Therefore a global search was run to select the 100 starting ligand conformations that yield the lowest energy function values for each protein. This resulted in one biased training dataset of 100 * 100 lowest energy ligand starting conformations and a second unbiased dataset of another 100 * 100 ligand starting conformations.

First we optimized the parameter σ of the SCG algorithm (Text S1) because there was no clear indication for its optimal value in the original paper [17]. We measured the average number of function and gradient evaluations the algorithm used in a local optimization run over the training sets described above, varying σ between 10^−13^ and 10^−5^ at the two convergence criteria ε_g_ = 10^−10^ and ε_g_ = 10^−5^. The results clearly indicate that the optimal value for σ is around 10^−9^ (Fig. S1), which is used for all subsequent tests. However, when the convergence criterion is not very strict, the algorithm is quite insensitive to the value of σ.

Next the competitive multistart (CMS) algorithm (Text S1) for global optimization was tested. We used the same training set of 100 ligand-protein complexes as before, but with randomized ligand conformations. Since the global energy function optimum of a ligand-protein complex is a priori unknown, we used the energy of the ligand protein crystal structure as the best available approximation. Further energy minimization by the local SCG algorithm with the strict convergence criterion ε_g_ = 10^−15^ yielded our best guess for the reference global energy optimum.

**Figure 1:**
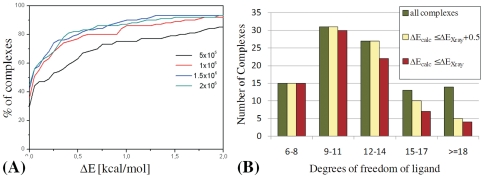
Performance and limitations of the global optimization algorithm CMS. (**A**) The number of test cases that were successfully optimized within a certain energy threshold from the reference value. On the left figure the reference value was the current estimate for the global optimum, and on the right it was the energy of the local optimized X-ray conformation of the ligand. (**B**) For all training set ligands with 6–14 degrees of freedom, the CMS algorithm finds an energy minimum within +0.5 kcal/mol of the crystallographic complex (ΔE_Xray_). For ligands with 15 or more degrees of freedom, the CMS minimization performance becomes worse than ΔE_Xray_+0.5 kcal/mol for an increasing number of complexes.

The CMS algorithm was run using the SCG method as the local optimization subroutine with σ = 10^−9^, ε_g_ = 10^−8^ and 0.5*10^6^, 1*10^6^, 1.5*10^6^ and 2*10^6^ function evaluations. Some ligand-protein complexes produced global energy minima below the reference optimum. In these cases our new estimate for the reference global optimum became this lower energy record. Comparing the results of the CMS global optimization to this reference optimum shows that the energies of the locally optimized ligand conformations are in about 80% of all test cases within 0.5 kcal/mol of the reference optimum (Fig. 1A). However, for ligands with 15 or more degrees of freedom the global search algorithm quickly drops in reliability (Fig. 1B), thereby limiting the application of FRIGATE to ligands with less than 15 to 18 degrees of freedom.

### Comparing FRIGATE to benchmark docking programs

**Figure 2:**
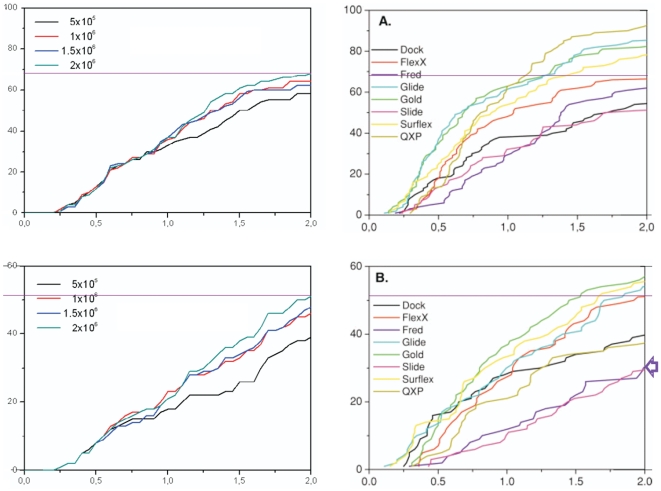
Docking (top) and scoring (bottom) accuracy of FRIGATE compared with benchmark programs [15]. (**A**) FRIGATE docks ∼68% (horizontal line) of the 30 top scoring ligand poses within 2 Å of the crystallographic pose, which matches the average performance of the benchmark programs. (**B**) FRIGATE scores ∼52% of the top scoring ligand poses within 2 Å of the crystallographic pose, which exceeds the performance of FRED1.1 (arrow) by ∼23%.

Two pivotal quality parameters for computational screening and docking programs are docking accuracy and scoring accuracy. Utilizing the aforementioned dataset of 100 ligand-protein crystal structures [15], we firstly energy minimized the crystallographic ligand conformer in vacuum in order to mimic virtual screening applications where the bound ligand conformer is unknown. Secondly we ran FRIGATE with 0.5*10^6^, 1*10^6^, 1.5*10^6^ and 2*10^6^ energy function evaluations for each ligand-protein complex and retained the 30 top scoring ligand poses for each complex. Next we counted all retained ligand poses that are found within a certain rmsd distance from the experimental X-ray pose [16]. Plotting this number of poses as a percentage of all complexes against a meaningful rmsd distance scale of 0 to 2 Å gives a graphical representation of the docking accuracy (Fig. 2A). When 2*10^6^ energy function evaluations are calculated FRIGATE poses 68% of the top 30 scoring ligands within 2 Å of the crystallized ligand, which is approximately as good as the mean docking accuracy of the eight benchmark programs assessed in a previous study [15].

The scoring accuracy plot (Fig. 2B) shows the percentage of all 100 ligand-protein complexes where the top scoring pose lies within a certain rsmd distance from the experimental pose. Again with 2*10^6^ energy function evaluations FRIGATE puts the top scoring pose in 52% of all complexes within 2 Å rsmd distance of the crystallized ligand. This scoring accuracy compares very well with the better half of the eight benchmark programs [15]. Taking into account that FRIGATE utilizes the unbiased energy minimized ligand conformer, suggests that the FRIGATE docks and scores ligands, whose bound conformation is unknown, comparatively accurately.

We hypothesize that the superior performance of FRIGATE stems from its two-step approach: firstly, FRIGATE mimics the random move of a ligand through the stochastic approach of CMS global optimization. Secondly, FRIGATE fine-tunes the global minimum by following the robust SCG energy optimization path of molecular dynamics. On average FRIGATE docks one ligand in 10–20 sec on a 64 bit Celeron Intel processor, providing sufficient speed for virtual screens of >1 Mio. ligands.

### FRIGATE identifies novel ligands to Ag85c

In this study we test FRIGATE on the potential anti-TB target protein Ag85C of *Mtb*, which appears well suited due to its deep TMM binding trough and the availability of two co-crystal structures with the substrate mimetics OTG and trehalose in the catalytic site. In addition FRIGATE covers potential allosteric ligand binding sites, thereby enabling the discovery of allosteric modulators which frequently go undetected in experimental or active site pharmacophore-based computational screens. As the ligand database for FRIGATE we used the 2,066,536 drug-like and Rule-of-5 compliant compounds of the ZINC library, which exclusively contains commercially available compounds for experimental follow-up [18]. The 3D ligand conformers were generated with the program Omega (OpenEye), and the partial charges were calculated with the semi-empirical quantum mechanical program AMSOL [19].

A second solubility-filtered ligand database was generated by removing molecules from the abovementioned ZINC set that are unlikely to be soluble in water. We kept only molecules with ClogP values of less than 3 or polar desolvation energies of less than −30 kJ/mol, resulting in a database of 1,035,013 molecules.

The FRIGATE screen of the 2,066,536-molecule unfiltered library was completed within less than 320 hours on a cluster of 48 processors, using the coordinates of the apo Ag85C crystal structure [20]. Hit compounds were rank ordered according to their computed docking energies by the FRIGATE program. Of the 100 top scoring VS hits, 96 compounds were docked into the deep catalytic site of Ag85C and merely 4 into a second shallow surface site between residues D44 and Y172. The clear preference for the catalytic site demonstrates that FRIGATE places hits into the most druggable site. However the docking scores of the 4 second site hits do not rank them to the bottom of top 100 list as estimated from the lower druggability of this site, but rather throughout the list at positions 11, 43, 51 and 86. Consequently FRIGATE does identify surface binders, which is useful for discovery programs on non-catalytic targets and allosteric sites, but its docking energies do not unambiguously direct one to the most druggable site.

### Experimental hit validation by NMR

The 60 top ranking FRIGATE hits from the solubility filtered library were manually checked for chemical attractiveness and commercial availability at reasonable cost, resulting in the acquisition of 23 compounds. Since less polar compounds may favorably interact with the sizable hydrophobic trough in the catalytic site of Ag85C and solubility filtering often suffers from false-positives, we additionally assessed the 60 top ranking VS hits from the unfiltered library. This led to another 8 compounds that were commercially available and chemically attractive (Table S1).

**Figure 3:**
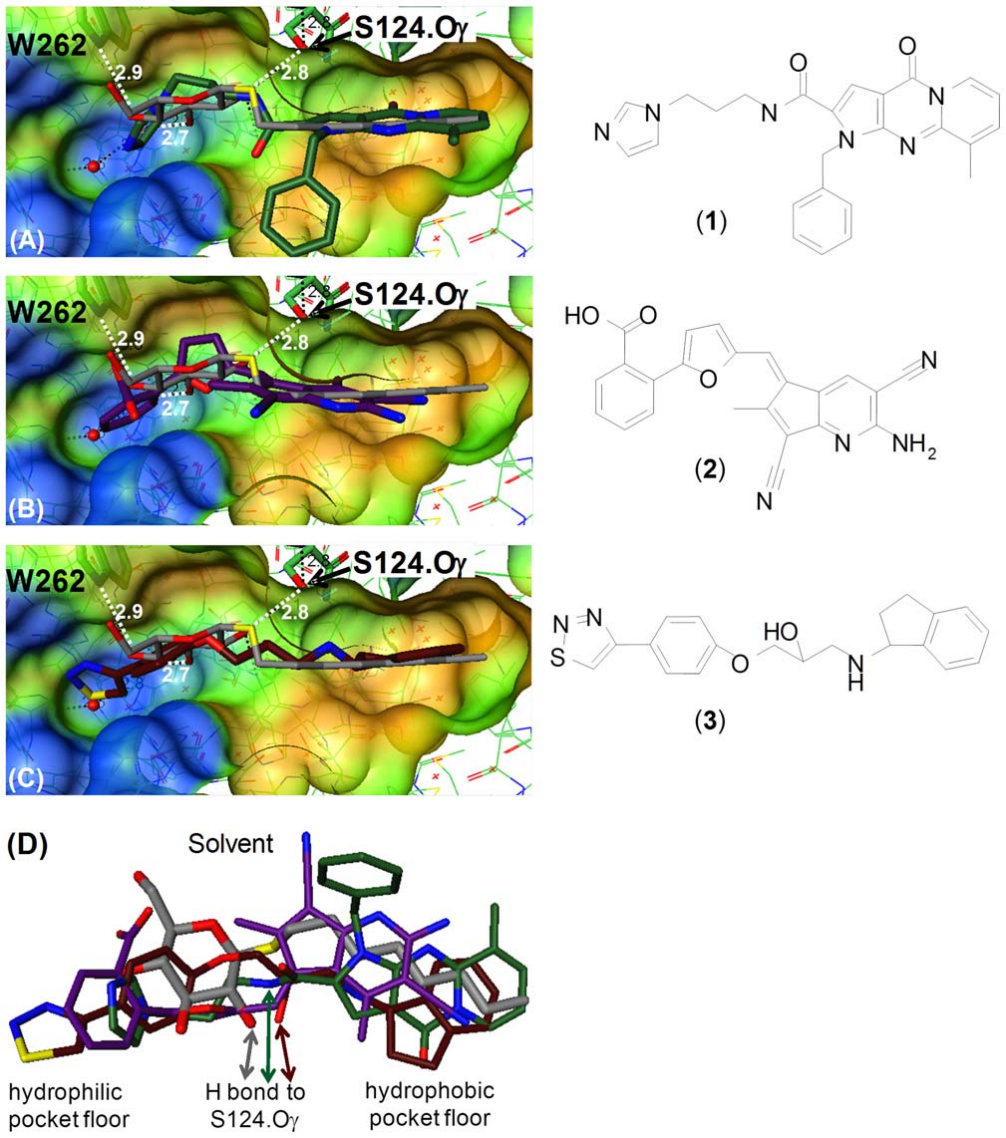
FRIGATE docking models of confirmed hits in the catalytic site of Ag85C. (**A**) **1** (C atoms dark green) superimposed on the co-crystal structure of OTG (grey) bound to Ag85C [9] using the protein coordinates for the overlay. H bonds between OTG and Ag85C are shown as dashed lines with distances in Å, including the catalytic oxyanion (Oγ) of S124. The semi-transparent surface of Ag85C is color coded from polar (blue) to hydrophobic (brown). (**B**) Superimposition of **2** (purple) (**C**) Superimposition of **3** (dark brown) (**D**) Superimposition of **1** to **3** onto OTG highlighting the differential pocket coverage. Ligand color-coding identical to (A)–(C).

**Figure 4:**
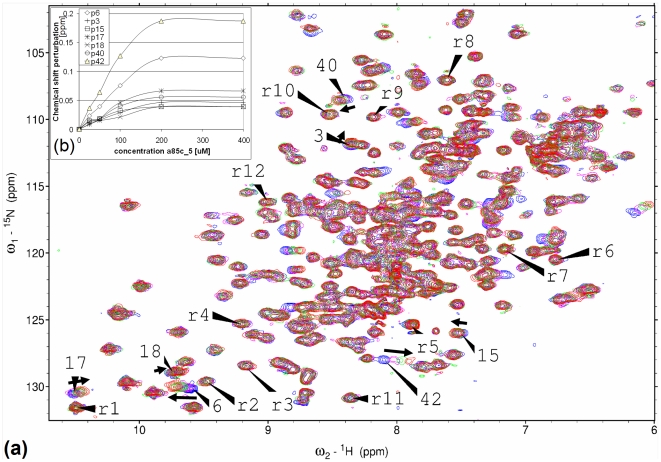
NMR binding assay of 1. (**a**) The ^15^N-HSQC spectra of Ag85C in the presence of 50 µM (purple), 100 µM (green) and 200 µM (red) **1** show mono-directional (arrows) CSPs for exemplary residues (numbered, eg 42) as compared with apo-Ag85C (blue). Twelve randomly selected reference residues (labeled r1–r12) remain unchanged. (**b**) Plotting the CSPs vs. the concentration of **1** allows one to fit a binding curve with an average Kd value of 177 µM. CSP numbering identical to (a).

The total of 31 compounds were tested for direct binding to Ag85C by NMR and for mycobacterial growth inhibition against *Mycobacterium smegmatis* (*Msmeg*). In the ^15^N-HSQC-NMR binding assay, compounds **1** and **2** (Fig. 3A+B) show strong chemical shift perturbations (CSPs) of the ^1^H-^15^N backbone amide signals of Ag85C (Fig. 4A), compound **3** (Fig. 3C) and two further compounds show weak CSPs, one compound shows protein precipitation, and the remaining 25 compounds are inactive (Table S1). CSPs are local, residue-based sensors for ligand – protein interactions or ligand-induced alterations in local protein conformation, and give a fingerprint of where the ligand binds to the protein [21]. We categorized the CSPs of this single concentration assay into “strong”, “weak” and “inactive” in comparison with two positive control ligands: the TMM mimetic n-octyl-thioglucoside (OTG), whose binding to the catalytic site of Ag85C is defined by a co-crystal structure [9], and the chemically unrelated fragment 2-aminocyclohepta[*b*]thiophene-3-carbonitrile (**5**) (Fig. S5), which was identified by experimental screening against a diverse fragment library [22].

### FRIGATE docking site confirms in NMR assay

Furthermore the CSPs were utilized to differentiate between ligand binding to the catalytic site and other sites of Ag85C. Comparing the CSPs between **1** and OTG shows that 20 out of a total of 31 CSPs correlate, as defined by being larger than the CSPs of 15 randomly selected reference peaks, which reflect the random variation of the assay (Fig. S2; Fig. S4A). Similarly, 21 of the same total of 31 CSPs correlate between **1** and **5** (Fig. S3; Fig. S4B). These data indicate that **1** binds to the same site as OTG or a directly adjacent sub-site in the catalytic cleft, which is in agreement with the docking model of **1** (Fig. 3B). A small number of divergent CSPs is within expectation because **1** and OTG as well as **1** and **5** fill different segments of the catalytic pocket and locally place variant ligand atoms in the vicinity of the protein ^15^NH groups.

Applying the same CSP correlation analysis to **2** yields 27 CSPs out of the total 31 CSPs that correlate between **2** and OTG (Fig. S4C), indicating binding the catalytic site, which again corroborates the docking model of **2** (Fig. 3B). The three weak binders merely show a fraction of the CSPs observed for OTG and **5**. Due to this lack of data points, binding site information could not be unambiguously extracted from the CSP data. Taken together, 5 out of 31 FRIGATE hits confirmed in the NMR binding assay, yielding a hit confirmation rate of 16%, which is within range of the 1 to 20% hit rates of published target-based virtual screening campaigns by other software programs [2], [23]. The two most active hits bind to the catalytic site of Ag85C, as predicted by FRIGATE and expected from the superior druggability of this site.

### Antibacterial activity testing

**Figure 5:**
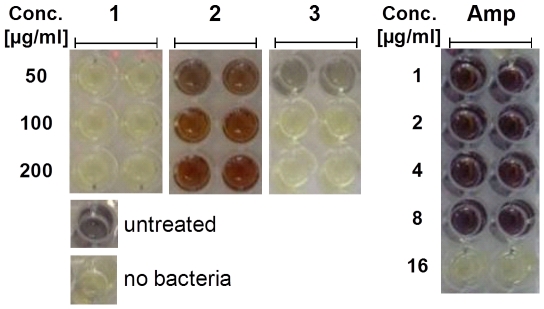
*Msmeg* growth inhibition assay of 1 to 3. Addition of 50 and 100 µg/mL of **1** and **3**, respectively, blocks the growth of *Msmeg* beyond the level of visible detection, whereas *Msmeg* grows in the presence of 50–200 µg/mL of **2**. The reference antibiotic ampicillin (Amp) inhibits *Msmeg* growth at 16 µg/mL as published previously [37]. Growth medium with (labeled ‘untreated’) and without bacteria inoculum (labeled ‘no bacteria’) is colored black and pale yellow, respectively.

Learning from the rich literature of potent in vitro enzyme inhibitors that were discarded due to the lack of antibacterial activity [24], we assayed the 31 selected FRIGATE hits for antibacterial activity prior to in vitro follow-up work. We utilized the less pathogenic and faster growing *Mtb* model organism *Msmeg* for primary growth inhibition testing. Of the total 31 FRIGATE hits, **1** shows the strongest anti-mycobacterial activity with a MIC value of 50 µg/mL or less (Fig. 5). Two further compounds **3** and **4** showed MIC values of 100 µg/mL, whereas the remaining 28 FRIGATE hits did not inhibit the growth of *Msmeg* at compound concentrations between 50 and 200 µg/mL (Table S1). Hence **1** exhibits the highest activity in both the Ag85C binding assay and the antibacterial *Msmeg* assay, while **3** is substantially less active in these assays and **2** and **4** lack either anti-mycobacterial or Ag85C binding activity. Since **4** was soluble in the NMR assay (Table S1), but did not show binding to Ag85C in the ^15^N-HSQC spectrum, it may inhibit *Msmeg* growth by a mechanism independent of Ag85C.

On the basis of this data, we prioritized **1** for follow-up assays. By 15N-HSQC-NMR titration experiments we determined a Kd value of 177 µM for the in vitro binding of **1** to Ag85C (Fig. 4b). Next we tested **1** against the MDR-*Mtb* strain 2745/09 in the radiometric BACTEC 460 assay in liquid broth [25]. **1** stopped the growth of this MDR-*Mtb* strain with a MIC of 20 µg/mL, which is in good agreement with its MIC against *Msmeg* of < = 50 µg/mL. The robust inhibitory effect on MDR-*Mtb* demonstrates that **1** acts through a mode-of-action (MOA) different from isoniazid and rifampicin, which define MDR-*Mtb* strains and block mycolyl synthesis via enoyl-ACP reductase and DNA-dependent RNA polymerase, respectively [26], [27]. Testing **1** for bacteriotoxicity yielded no bactericidal activity against MDR-*Mtb* up to a concentration of 80 µg/mL, which is not unexpected at this low level of potency. In summary **1** blocks the growth of *Msmeg* and MDR-*Mtb* with similar potency and shows potential for combating the surge of MDR-*Mtb* infections.

### Confirmed ligands show diverse pocket fill

The FRIGATE docking models of the experimentally validated hits **1**, **2** and **3** reveal that they converge in occupying most of the central pocket as defined by OTG co-crystallized with Ag85C (Fig. 3A–C). However, at both termini of the pocket they diverge in the level of occupancy. **1** fills the hydrophobic sub-pocket more thoroughly than **2** and **3**, and penetrates deeper into this sub-pocket than OTG (Fig. 3D), even though the carbonyl on the tricycle of **1** may not be the most favorable functionality for this lipophilic protein site lacking H bond donor counter-parts. In the hydrophilic sub-pocket, **1** occupies a similar space as OTG, where it mediates an H bond between its imidazole π electron cloud and the NHε of W262 according to our model. OTG also forms an H bond with W262.NHε through the O atom of its 4′ hydroxy group.

**2** shows the least hydrophobic sub-pocket coverage, but possesses more atoms interacting with the central and hydrophilic sub-pockets. Its highly solvated carboxylate and amino moieties stretch away from the protein into solvent (Fig. 3D), which may favorably orient **2** for binding. In contrast to **1** and **3**, **2** lacks the prominent H bond contact with the catalytic oxyanion of S124, a pivotal interaction conserved among all ligands co-crystallized with Ag85 enzymes so far [8], [9], [20]. In effect **2** does not mediate any of the three H bond contacts observed between the glucoside of OTG and Ag85C in the co-crystal, even though the carboxylate of **2** is within close 2.9 Å distance to the W262.NHε of Ag85C.

**3** extends the furthest into the hydrophilic sub-pocket, thereby replacing water molecule 2025 (Fig. 3C), which is well coordinated by the backbone amides of Q43, Y46 and G48 and the carboxylate of D38. Since water 2025 is conserved in all of the crystal structures of Ag85A, B and C, its replacement may be energetically costly. Also the secondary amino group of **3** may be a less preferred functionality in the hydrophobic sub-pocket, as suggested by the SAR data from analogues of **5**, where a tertiary carbon to nitrogen substitution in this segment of the pocket led to inactivity [22].

Although these 3D docking models have not been experimentally validated by crystal structures and may be incorrect, three common themes can be deduced. Firstly, the central pocket segment is filled by diverse chemical matter. Secondly, the occupancy of both terminal segments of the pocket varies. Thirdly, the number of H bond contacts in the glucoside binding sub-pocket as well as a few contacts in the hydrophobic sub-pocket appear to be sub-optimal with respect to the interactions observed with OTG.

### Ligand efficiency benchmarking

The identification of diverse hit chemical matter and variant ligand placement across the entire pocket is highly desirable for synthetic library follow-up because chemical series with orthogonal safety profiles can be pursued and hybrid design approaches are enabled. However sub-optimal ligand - protein interactions are a serious disadvantage, which may necessitate laborious subtractive chemistry or even high-risk scaffold hopping. To explore the protein interaction quality of **1** in more detail, we utilize the ligand efficiency parameter, BEI, defined as –log_10_(Kd or IC_50_ or MIC [mol/L])/(Mw [kDa]) [28]. Using its Kd value in the NMR binding assay, an in vitro ligand binding efficiency, BEI, of 8.5 is calculated for **1**, which is substantially lower than the BEI of 20, measured for the Ag85C ligand **5** in the same NMR-based Kd assay [22]. Since marketed small molecule drugs for druggable enzymes, such as protein kinase inhibitors, possess IC_50_-based BEI values of 17 to 20 [29], substantial synthetic optimization of **1** seems mandatory.

With the goal of therapeutic usage, anti-mycobacterial ligand efficiency is even more relevant. Calculated from its MIC against MDR-*Mtb*, **1** possesses an anti-tubercular BEI of 9.9, which is in line with the *Msmeg* MIC-based BEI of 9.0 of synthetic phosphonate and sulfonate inhibitors of Ag85C, which were designed to mimic the enzymatic transition state [30], [31]. A similar anti-mycobacterial BEI of 9.0 is calculated for the covalent Ag85C inhibitor 6-azido-6′-deoxytrehalose from its MIC against *M. aurum*. However, compared with the fragment-derived analogue of **5**, 2-amino-6-propyl-cyclohexa[*b*]thiophene-3-carbonitrile (**6**), with MIC-based BEI values of 17 to 18 against MDR-*Mtb* [22], **1** again underperforms. Benchmarking **1** against the most advanced clinical phase anti-tuberculars, moxifloxacin (clinical phase III), TMC207, PA-824 and OPC-67683 (all clinical phase II) with MIC-based BEI values of 15, 13, 17 and 14 against *Mtb* strain H37R, respectively, underlines the notion that **1** needs synthetic optimization with respect to both ligand efficiency and absolute potency towards a MIC of 0.5 µg/mL or lower [4], [32], [33].

In conclusion FRIGATE rapidly produced chemically diverse hits for all parts of the catalytic pocket of Ag85C. One FRIGATE hit, **1**, was experimentally confirmed to bind to the catalytic site of Ag85C and inhibits the growth of *Msmeg* and MDR-*Mtb* with a robust MIC of 20 µg/mL. Although **1** does not match the in vitro binding efficiency and *Mtb* growth inhibition efficiency of the Ag85C ligand **6** and clinical development candidates blocking other targets of *Mtb*, **1** is a good starting point for synthetic optimization towards both improved target binding and anti-tubercular potency. While most published virtual screens fail to report hits with anti-bacterial activity [24], [34], [35], [36], the discovery of **1** shows that FRIGATE can quickly and cost-efficiently generate hits with biological activity and is an attractive tool for organizations that lack extensive compound screening infrastructure.

## Methods

(see Text S1)

## Supporting Information

Figure S1 **Parameterization of the the SCG local optimization algorithm.** The average number of function and gradient evaluations needed for the SCG algorithm shows a minimum at σ = 10^−9^ with a high quality convergence criterion of ε_g_ = 10^−10^, but not with a low quality ε_g_ = 10^−5^. This finding holds true for both the unbiased (left) and biased (right) training set.
(TIF)

Figure S2 **The overlay of the 15N-HSQC spectra of 50 µM 15N-labeled Ag85C alone (blue) and Ag85C in the presence of 400 µM 1 (red) and 550 µM OTG (green) shows several CSPs common between 1 and OTG, such as those labeled 12, 17, 19, 40 and 44 (arrows).** The 15 reference resonances (labeled ‘r1’ to ‘r15’) remain virtually unchanged.
(TIF)

Figure S3 **The overlay of the ^15^N-HSQC spectra of 50 µM ^15^N-labeled Ag85C alone (blue) and Ag85C in the presence of 400 µM 1 (red) and 400 µM 5 (green) shows several CSPs common between 1 and 5, such as those labeled 12, 17, 19, 40 and 44 (arrows).** The 15 reference resonances (labeled ‘r1’ to ‘r15’) remain virtually unchanged.
(TIF)

Figure S4 **31 resolved CSPs (filled rhombs) and 15 reference resonances (open circles) from the ^15^N-HSQC spectrum with OTG are plotted against the same CSPs and reference resonances in the ^15^N-HSQC spectrum in the presence of 1 or 2.** (**A**) 20 out of the total 31 CSPs correlate between **1** and OTG in showing mutual CSPs larger than the variation of reference resonances. (**B**) 21 out of the total 31 CSPs correlate between **1** and **5**. (**C**) 27 out of the total 31 CSPs correlate between **2** and OTG.
(TIF)

Figure S5 **Chemical structures of 5 and 6.**
(TIF)

Table S1 **Ag85C binding and** ***Msmeg*** **antibacterial activity of the selected 31 FRIGATE hits, sorted by decreasing FRIGATE score from top to bottom.**
(DOC)

Text S1 **NMR binding site and antibacterial assay results for the abovementioned compounds, experimental methods, description of computational algorithms.**
(DOC)

## References

[1] De Azevedo WF (2010). MolDock applied to structure-based virtual screening.. Curr Drug Targets.

[2] Villoutreix BO, Eudes R, Miteva MA (2009). Structure-based virtual ligand screening: recent success stories.. Comb Chem High Throughput Screen.

[3] Young DB, Perkins MD, Duncan K, Barry CE (2008). Confronting the scientific obstacles to global control of tuberculosis.. J Clin Invest.

[4] Ginsberg AM (2008). Emerging Drugs for Tuberculosis.. Seminars Respirat Crit Care Medicine.

[5] Kremer L, Dover LG, Carrere S, Nampoothiri KM, Lesjean S (2002). Mycolic acid biosynthesis and enzymic characterization of the beta-ketoacyl-ACP synthase A-condensing enzyme from Mycobacterium tuberculosis.. Biochem J.

[6] Brennan PJ, Nikaido H (1995). The envelope of mycobacteria.. Annu Rev Biochem.

[7] Belisle JT, Vissa VD, Sievert T, Takayama K, Brennan PJ (1997). Role of the major antigen of Mycobacterium tuberculosis in cell wall biogenesis.. Science.

[8] Anderson DH, Harth G, Horwitz MA, Eisenberg D (2001). An interfacial mechanism and a class of inhibitors inferred from two crystal structures of the Mycobacterium tuberculosis 30 kDa major secretory protein (Antigen 85B), a mycolyl transferase.. J Mol Biol.

[9] Ronning DR, Vissa V, Besra GS, Belisle JT, Sacchettini JC (2004). Mycobacterium tuberculosis antigen 85A and 85C structures confirm binding orientation and conserved substrate specificity.. J Biol Chem.

[10] Jackson M, Raynaud C, Laneelle MA, Guilhot C, Laurent-Winter C (1999). Inactivation of the antigen 85C gene profoundly affects the mycolate content and alters the permeability of the Mycobacterium tuberculosis cell envelope.. Mol Microbiol.

[11] Harth G, Horwitz MA, Tabatadze D, Zamecnik PC (2002). Targeting the Mycobacterium tuberculosis 30/32-kDa mycolyl transferase complex as a therapeutic strategy against tuberculosis: Proof of principle by using antisense technology.. Proc Natl Acad Sci U S A.

[12] Morris GM, Goodsell DS, Halliday RS, Huey R, Hart WE (1998). Automated Docking Using a Lamarckian Genetic Algorithm and an Empirical Binding Free Energy Function.. J Comput Chem.

[13] Szabadka Z (2007). Predicting Protein-Ligand Binding by Molecular Docking, Ph.D.Thesis Grolmusz V, editor.

[14] Steihaug T (1983). The Conjugate Gradient Method and Trust Regions in Large Scale Optimization.. SIAM Journal on Numerical Analysis.

[15] Kellenberger E, Rodrigo J, Muller P, Rognan D (2004). Comparative evaluation of eight docking tools for docking and virtual screening accuracy.. Proteins.

[16] Paul N, Rognan D (2002). ConsDock: A new program for the consensus analysis of protein-ligand interactions.. Proteins.

[17] Moller M (1993). A scaled conjugate gradient algorithm for fast supervised learning.. Neural Networks.

[18] Irwin JJ, Shoichet BK (2005). ZINC–a free database of commercially available compounds for virtual screening.. J Chem Inf Model.

[19] Hawkins GD, Giesen DJ, Lynch GC, Chambers CC, Rossi I (2003). AMSOL- version 7.0.

[20] Ronning DR, Klabunde T, Besra GS, Vissa VD, Belisle JT (2000). Crystal structure of the secreted form of antigen 85C reveals potential targets for mycobacterial drugs and vaccines.. Nat Struct Biol.

[21] Schade M (2006). NMR fragment screening: Advantages and applications.. IDrugs.

[22] Scheich C, Puetter V, Schade M (2010). Novel Small Molecule Inhibitors of MDR Mycobacterium tuberculosis by NMR Fragment Screening of Antigen 85C.. J Med Chem.

[23] Senderowitz H, Marantz Y (2009). G Protein-Coupled Receptors: target-based in silico screening.. Curr Pharm Des.

[24] Payne DJ, Gwynn MN, Holmes DJ, Pompliano DL (2007). Drugs for bad bugs: confronting the challenges of antibacterial discovery.. Nat Rev Drug Discov.

[25] Pfyffer GE, Bonato DA, Ebrahimzadeh A, Gross W, Hotaling J (1999). Multicenter laboratory validation of susceptibility testing of Mycobacterium tuberculosis against classical second-line and newer antimicrobial drugs by using the radiometric BACTEC 460 technique and the proportion method with solid media.. J Clin Microbiol.

[26] Vilcheze C, Jacobs WRJ (2007). The Mechanism of Isoniazid Killing: Clarity Through the Scope of Genetics.. Annu Rev Microbiol.

[27] Coulson CJ, Coulson CJ (1994). Bacterial RNA-Polymerase - Rifampin as Antimycobacterial.. Molecular Mechanisms of Drug Action.

[28] Abad-Zapatero C, Metz JT (2005). Ligand efficiency indices as guideposts for drug discovery.. Drug Discov Today.

[29] Hajduk PJ (2006). Fragment-based drug design: how big is too big?. J Med Chem.

[30] Gobec S, Plantan I, Mravljak J, Svajger U, Wilson RA (2007). Design, synthesis, biochemical evaluation and antimycobacterial action of phosphonate inhibitors of antigen 85C, a crucial enzyme involved in biosynthesis of the mycobacterial cell wall.. Eur J Med Chem.

[31] Kovac A, Wilson RA, Besra GS, Filipic M, Kikelj D (2006). New lipophilic phthalimido- and 3-phenoxybenzyl sulfonates: inhibition of antigen 85C mycolyltransferase activity and cytotoxicity.. J Enzyme Inhib Med Chem.

[32] Shandil RK, Jayaram R, Kaur P, Gaonkar S, Suresh BL (2007). Moxifloxacin, ofloxacin, sparfloxacin, and ciprofloxacin against Mycobacterium tuberculosis: evaluation of in vitro and pharmacodynamic indices that best predict in vivo efficacy.. Antimicrob Agents Chemother.

[33] Matsumoto M, Hashizume H, Tomishige T, Kawasaki M, Tsubouchi H (2006). OPC-67683, a nitro-dihydro-imidazooxazole derivative with promising action against tuberculosis in vitro and in mice.. PLoS Med.

[34] Talukdar A, Morgunova E, Duan J, Meining W, Foloppe N (2010). Virtual screening, selection and development of a benzindolone structural scaffold for inhibition of lumazine synthase.. Bioorg Med Chem.

[35] Lin TW, Melgar MM, Kurth D, Swamidass SJ, Purdon J (2006). Structure-based inhibitor design of AccD5, an essential acyl-CoA carboxylase carboxyltransferase domain of Mycobacterium tuberculosis.. Proc Natl Acad Sci U S A.

[36] Cosconati S, Hong JA, Novellino E, Carroll KS, Goodsell DS (2008). Structure-based virtual screening and biological evaluation of Mycobacterium tuberculosis adenosine 5′-phosphosulfate reductase inhibitors.. J Med Chem.

[37] Stephan J, Mailaender C, Etienne G, Daffe M, Niederweis M (2004). Multidrug resistance of a porin deletion mutant of Mycobacterium smegmatis.. Antimicrob Agents Chemother.

