# Supplementary figures and images for: Discovery of Novel MDR-Mycobacterium tuberculosis Inhibitor by New FRIGATE Computational Screen
Source: PLoS One. 2011 Dec 2;6(12):e28428. doi: 10.1371/journal.pone.0028428 (PMC3229595; doi:10.1371/journal.pone.0028428)

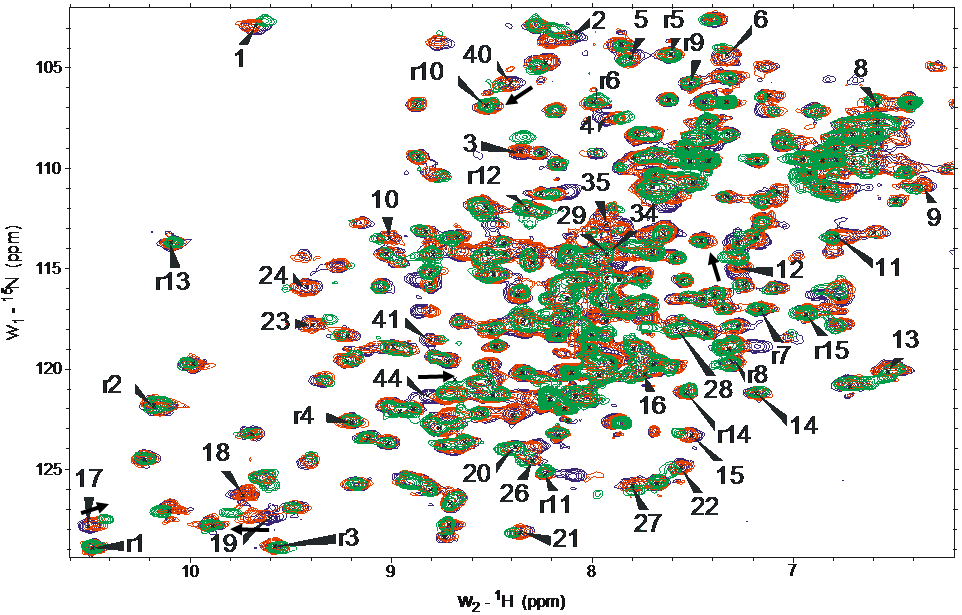

Supplement: Figure S2 — The overlay of the 15N-HSQC spectra of 50 µM 15N-labeled Ag85C alone (blue) and Ag85C in the presence of 400 µM 1 (red) and 550 µM OTG (green) shows several CSPs common between 1 and OTG, such as those labeled 12, 17, 19, 40 and 44 (arrows). The 15 reference resonances (labeled ‘r1’ to ‘r15’) remain virtually unchanged. (TIF) [file pone.0028428.s002.tif]

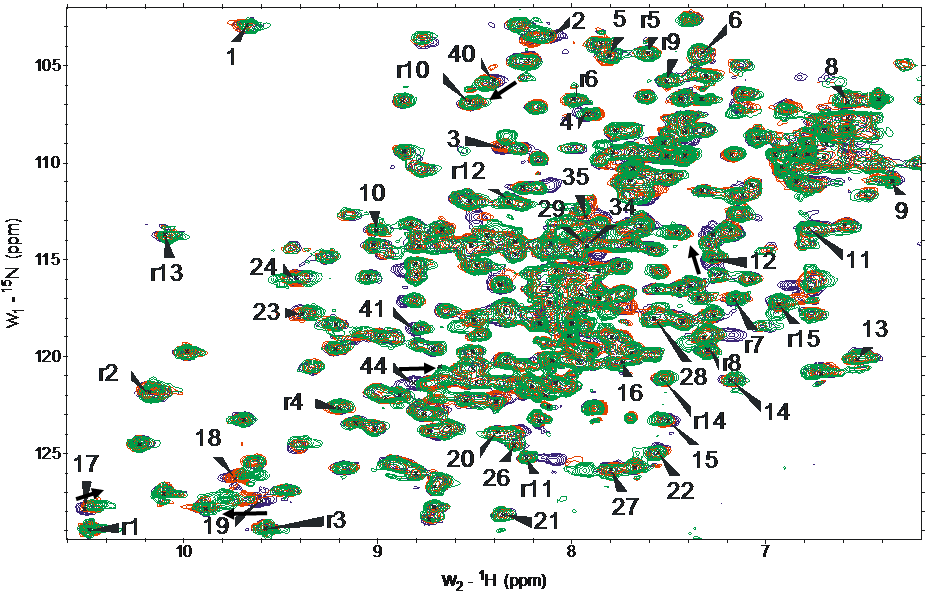

Supplement: Figure S3 — The overlay of the 15N-HSQC spectra of 50 µM 15N-labeled Ag85C alone (blue) and Ag85C in the presence of 400 µM 1 (red) and 400 µM 5 (green) shows several CSPs common between 1 and 5, such as those labeled 12, 17, 19, 40 and 44 (arrows). The 15 reference resonances (labeled ‘r1’ to ‘r15’) remain virtually unchanged. (TIF) [file pone.0028428.s003.tif]

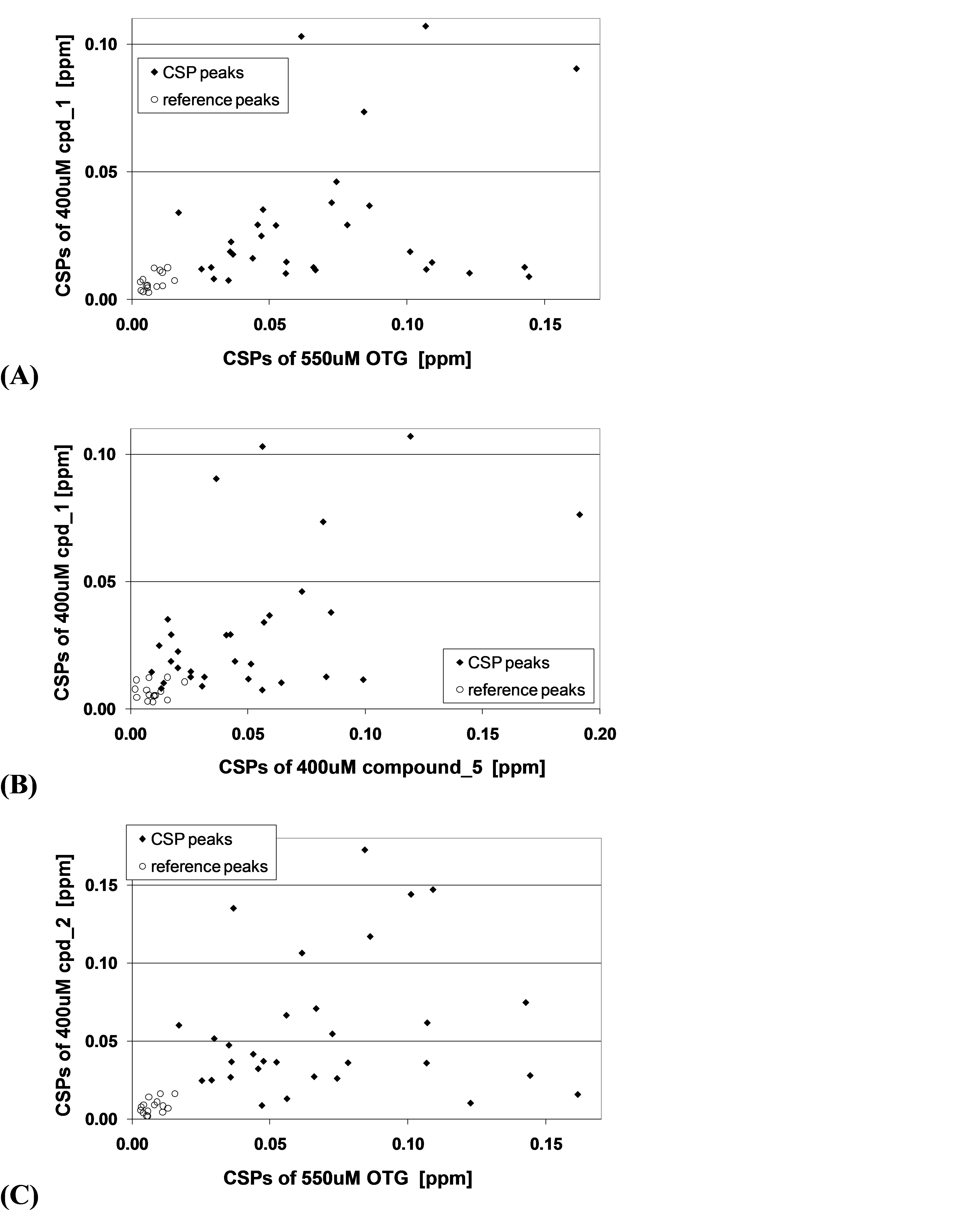

Supplement: Figure S4 — 31 resolved CSPs (filled rhombs) and 15 reference resonances (open circles) from the 15N-HSQC spectrum with OTG are plotted against the same CSPs and reference resonances in the 15N-HSQC spectrum in the presence of 1 or 2. (A) 20 out of the total 31 CSPs correlate between 1 and OTG in showing mutual CSPs larger than the variation of reference resonances. (B) 21 out of the total 31 CSPs correlate between 1 and 5. (C) 27 out of the total 31 CSPs correlate between 2 and OTG. (TIF) [file pone.0028428.s004.tif]

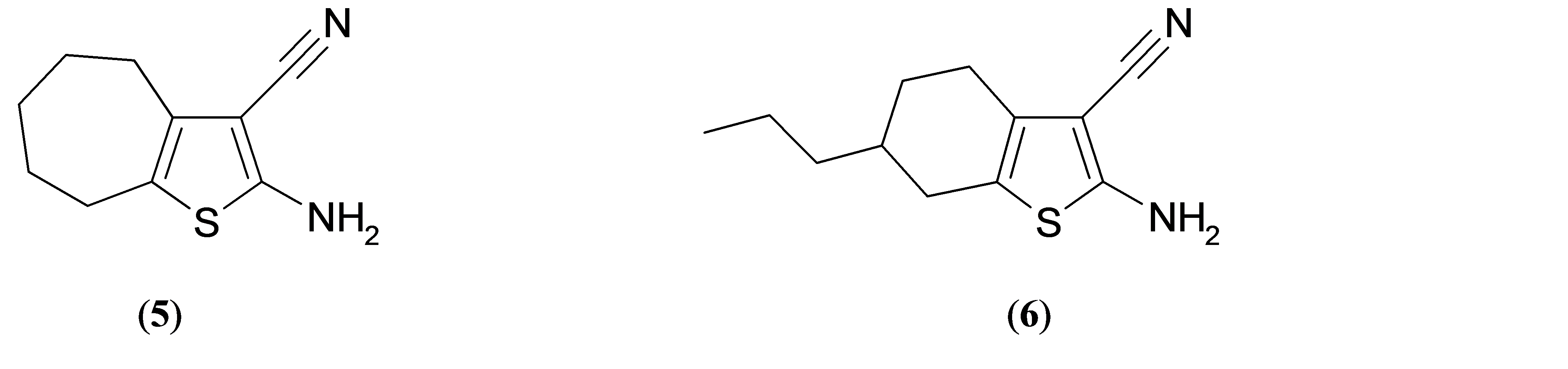

Supplement: Figure S5 — Chemical structures of 5 and 6. (TIF) [file pone.0028428.s005.tif]
