# Supplementary material for: Discovery of Novel MDR-Mycobacterium tuberculosis Inhibitor by New FRIGATE Computational Screen
Source: PLoS One. 2011 Dec 2;6(12):e28428. doi: 10.1371/journal.pone.0028428 (PMC3229595; doi:10.1371/journal.pone.0028428)
Supplement: Table S1 — Ag85C binding and Msmeg antibacterial activity of the selected 31 FRIGATE hits, sorted by decreasing FRIGATE score from top to bottom. (DOC) [file pone.0028428.s006.doc]

| Cpd No | Compound supplier: identifier | Mw [Da] | Solubility-filtered? | *Msmeg* growth inhibition [MIC ug/mL] | Binding to Ag85C by 15N-HSQC NMR | Solubility in 15N-HSQC-NMR assay by 1H-NMR | Binding site by FRIGATE | Binding site by 15N-HSQC-NMR |
| --- | --- | --- | --- | --- | --- | --- | --- | --- |
| **5** | positive control ligand | 192 |  | 64 | strong | - | cat | n.d. |
|  | ChemDiv: K279-0134 | 500 | - | >200 | no | n.d. | cat | n.d. |
|  | IBScreen: STOCK1S-72458 | 475 | yes | >200 | weak | soluble | cat | n.d. |
|  | IBScreen: STOCK3S-03608 | 492 | - | >200 | no | soluble | cat | n.d. |
| **1** | IBScreen: STOCK5S-42639 | 441 | yes | <50 | strong | soluble | cat | cat |
|  | Enamine: T0510-1928 | 446 | - | >200 | no | n.d. | cat | n.d. |
|  | Asinex: ASN05298059 | 462 | yes | >200 | weak | n.d. | 2nd site | n.d. |
| **4** | Enamine: T5265460 | 490 | - | 100 | no | soluble | cat | n.d. |
|  | Asinex: ASN04392302 | 457 | - | >200 | no | n.d. | cat | n.d. |
|  | Enamine: T0509-9019 | 478 | - | >200 | no | n.d. | cat | n.d. |
| **2** | IBScreen: STOCK5S-02830 | 407 | - | >200 | strong | soluble | cat | cat |
|  | Asinex: ASN05297954 | 476 | - | >200 | no | n.d. | cat | n.d. |
|  | ChemDiv: K788-5014 | 461 | yes | >200 | no | n.d. | cat | n.d. |
|  | IBScreen: STOCK5S-35204 | 458 | yes | >200 | no | soluble | cat | n.d. |
|  | Asinex: ASN10028063 | 422 | yes | >200 | no | soluble | cat | n.d. |
|  | Asinex: ASN04369766 | 484 | yes | >200 | no | soluble | 2nd site | n.d. |
|  | Enamine: ZU-4280644 | 451 | yes | >200 | no | soluble | cat | n.d. |
|  | Enamine: ZU-2607433 | 440 | yes | >200 | no | n.d. | cat | n.d. |
|  | Asinex: ASN05701949 | 477 | yes | >200 | no | soluble | 2nd site | n.d. |
|  | IBScreen: STOCK4S-67458 | 410 | yes | >200 | no | soluble | cat | n.d. |
|  | Enamine: T5305594 | 467 | yes | >200 | no | soluble | cat | n.d. |
|  | ChemDiv: K623-0513 | 493 | yes | >200 | no | soluble | cat | n.d. |
|  | Asinex: ASN05298102 | 482 | yes | >200 | no | n.d. | cat | n.d. |
|  | Enamine: T0509-0278 | 444 | yes | >200 | no | soluble | cat | n.d. |
|  | Enamine: T0518-0520 | 480 | yes | >200 | precipitate | soluble | cat | n.d. |
|  | Asinex: ASN03067791 | 402 | yes | >200 | no | n.d. | cat | n.d. |
|  | Enamine: ZU-0188280 | 414 | yes | >200 | no | soluble | cat | n.d. |
|  | ChemDiv: C060-0068 | 403 | yes | >200 | no | n.d. | cat | n.d. |
|  | Asinex: BAS07464421 | 395 | yes | >200 | no | soluble | cat | n.d. |
|  | Asinex: ASN04369422 | 490 | yes | >200 | no | soluble | cat | n.d. |
|  | Enamine: T0505-4374 | 367 | yes | >200 | no | soluble | cat | n.d. |
| **3** | Maybridge: HTS03852 | 369 | yes | 100 | weak | soluble | cat | n.d. |

n.d. = not detectable; precipitate = protein precipitates in presence of compound; cat = catalytic site of Ag85C; 2^nd^ site = surface site between D44 and Y172 of Ag85C
