# Supplementary material for: Discovery of Novel MDR-Mycobacterium tuberculosis Inhibitor by New FRIGATE Computational Screen
Source: PLoS One. 2011 Dec 2;6(12):e28428. doi: 10.1371/journal.pone.0028428 (PMC3229595; doi:10.1371/journal.pone.0028428)
Supplement: Text S1 — NMR binding site and antibacterial assay results for the abovementioned compounds, experimental methods, description of computational algorithms. (DOC) [file pone.0028428.s007.doc]

*Supporting Information*

Discovery of novel MDR-*Mycobacterium tuberculosis* inhibitor by new FRIGATE computational screen

Christoph Scheich, Zoltán Szabadka, Beáta Vértessy, Vera Puetter, Vince Grolmusz, Markus Schade

**Table of Contents**

[Scaled Conjugate Gradient (SCG) Algorithm 2](#__RefHeading___Toc289088740)

[Competitive MultiStart Algorithm 5](#__RefHeading___Toc289088741)

[Binding Efficiency Index (BEI) 6](#__RefHeading___Toc289088742)

[Results 6](#__RefHeading___Toc289088743)

[**Figure S1.** 6](#__RefHeading___Toc289088744)

[**Table S1**. 7](#__RefHeading___Toc289088745)

[**Figure S2.** 8](#__RefHeading___Toc289088746)

[**Figure S3.** 9](#__RefHeading___Toc289088747)

[**Figure S4.**. 10](#__RefHeading___Toc289088748)

[**Figure S5.**. 11](#__RefHeading___Toc289088749)

[Experimental Section 11](#__RefHeading___Toc289088750)

[Supporting References 11](#__RefHeading___Toc289088751)

# Scaled Conjugate Gradient (SCG) Algorithm

For local optimizations we used the Scaled Conjugate Gradient method.^1^


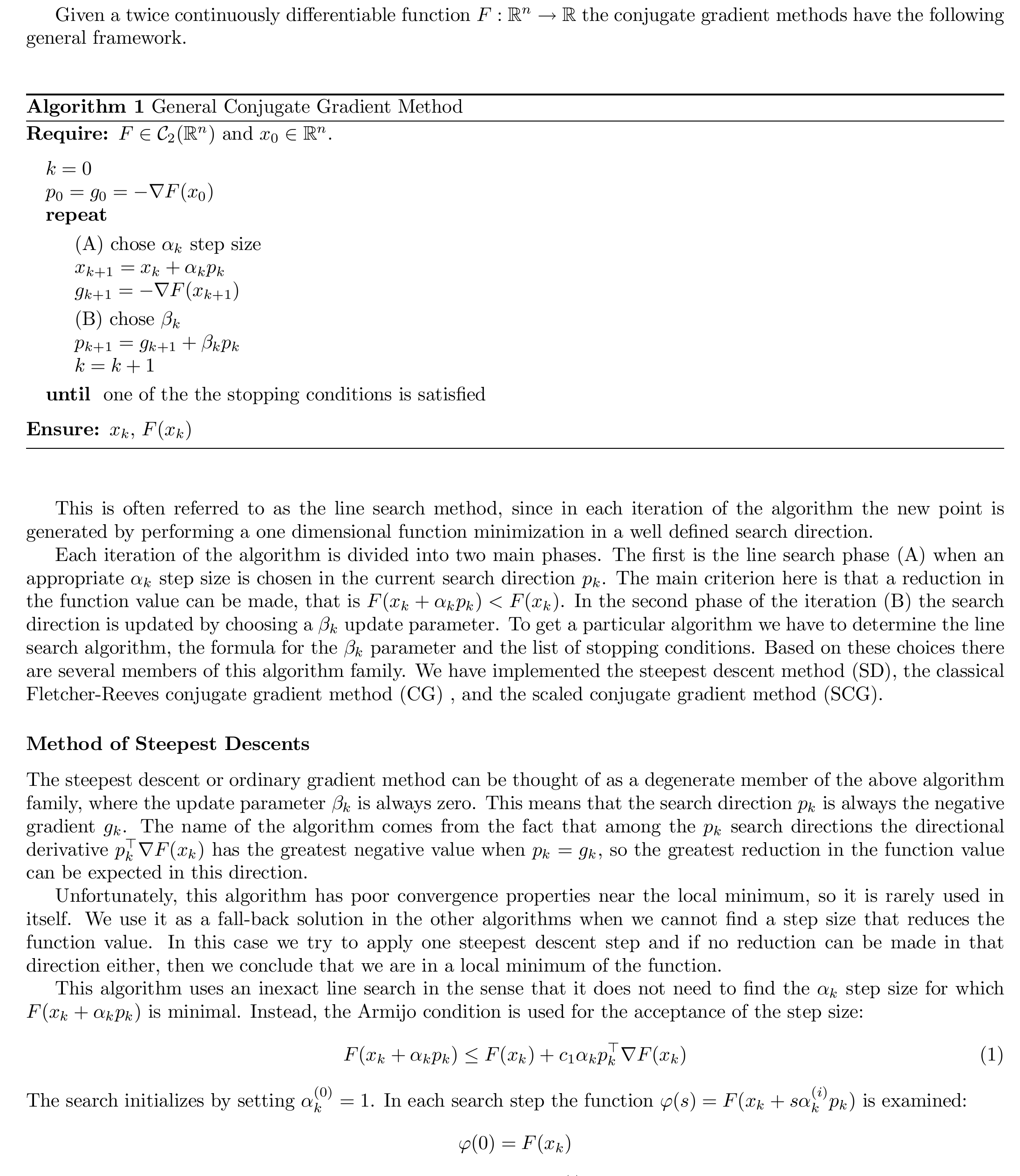


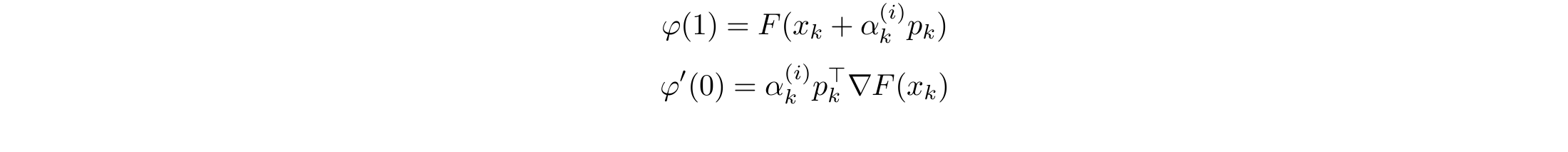


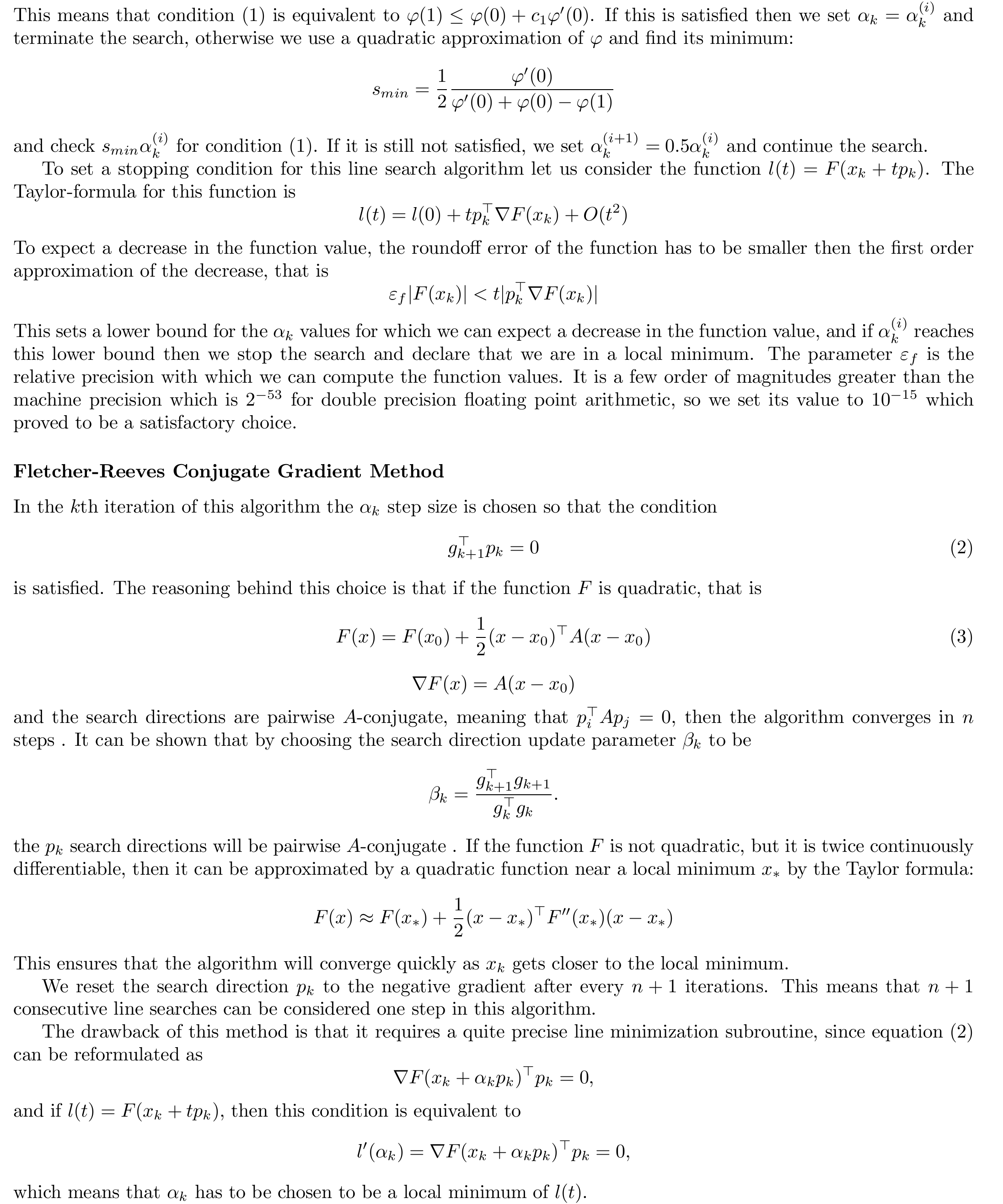


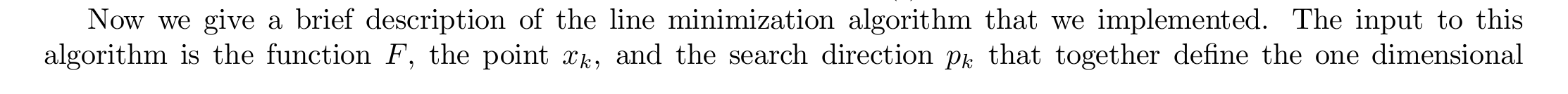


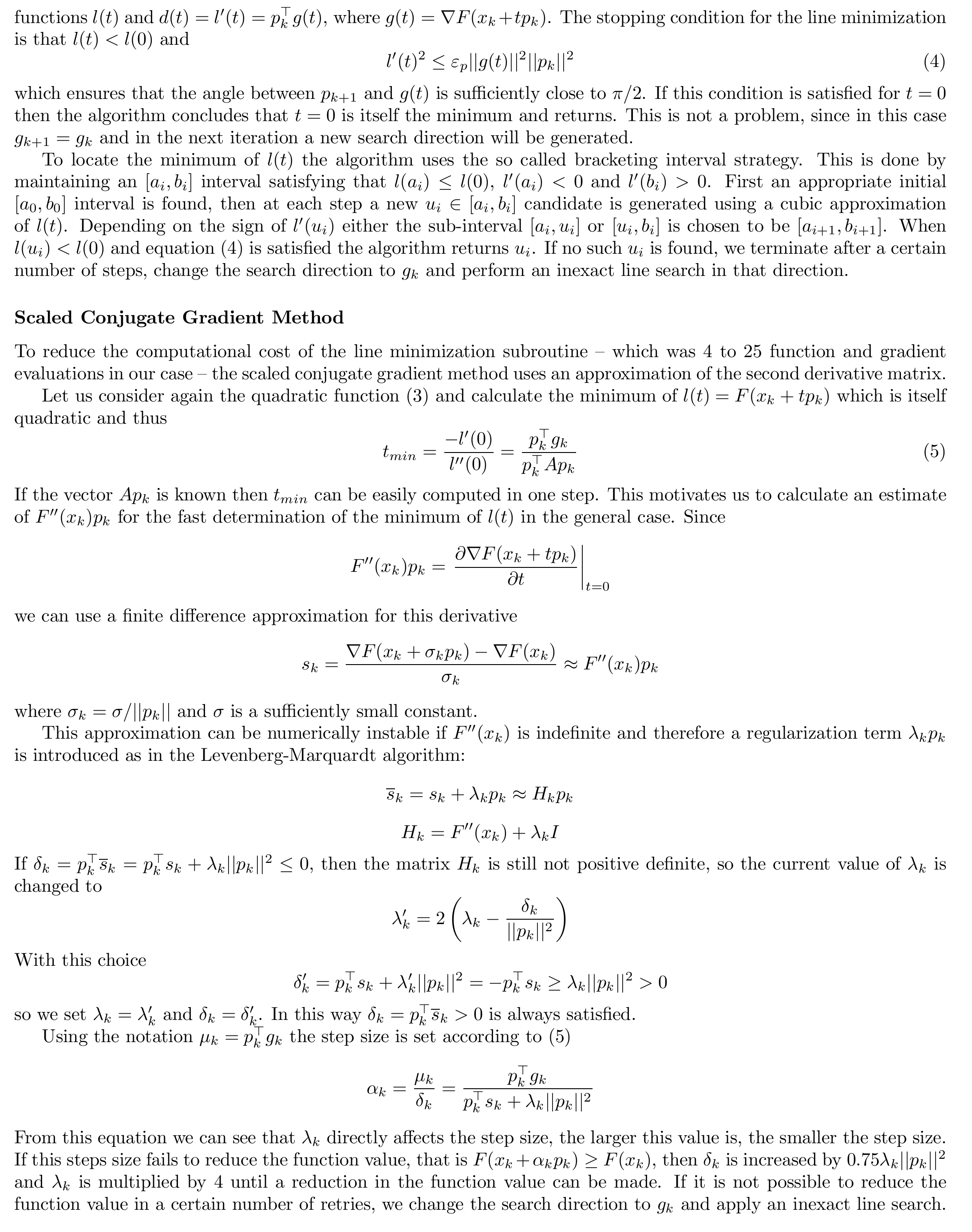


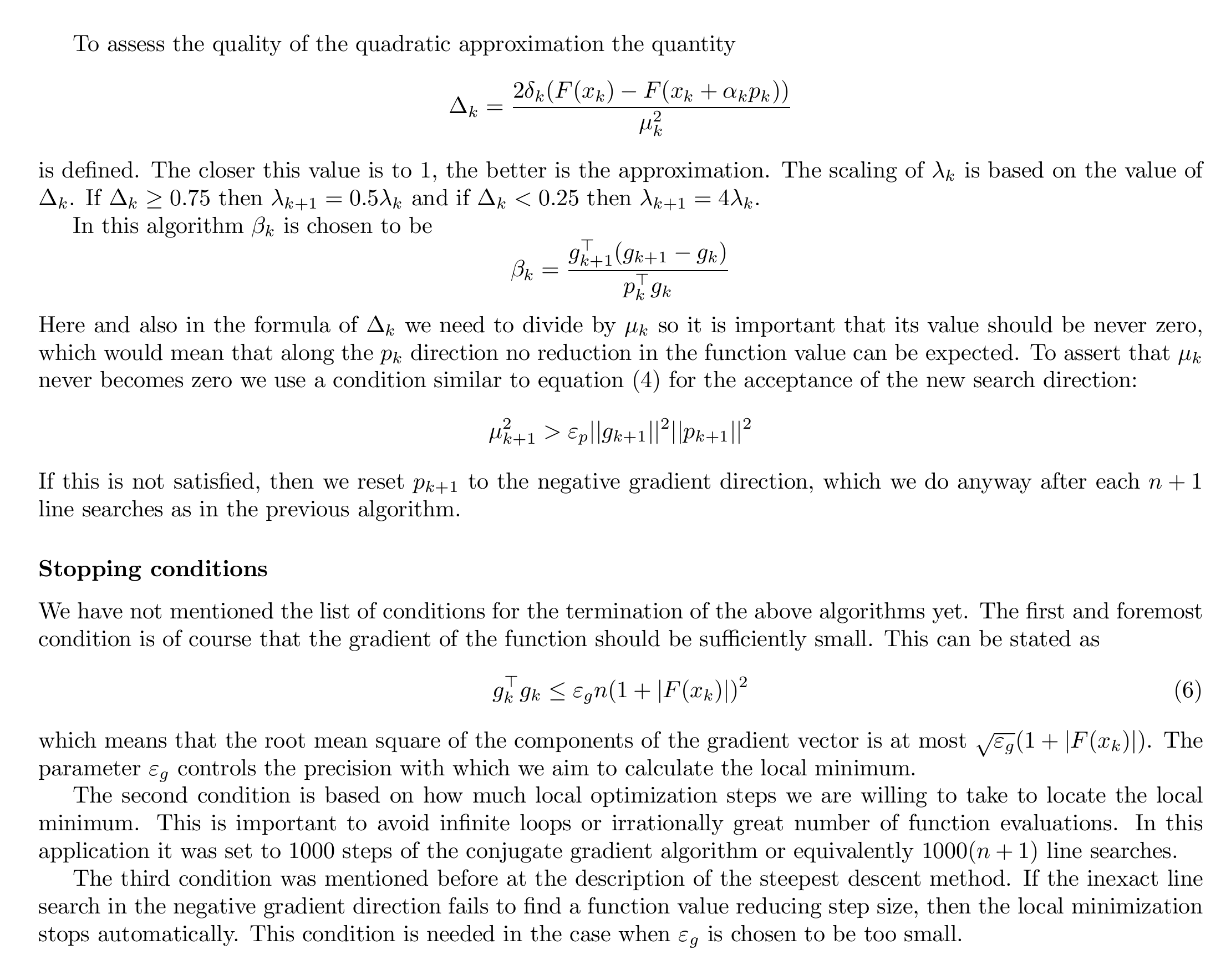


# Competitive MultiStart Algorithm

Generally the objective function to be optimized has many local minima and we have to find the one with lowest function value. One straightforward method is to start the local optimization from numerous randomly chosen starting points and approximate the global optimum by the lowest local minimum found this way. It is called the MultiStart algorithm*.^2^*

This method can be improved with a simple heuristics: we randomly choose the starting points but instead of finding the local minimum we apply only *n* line searches on each of them first. After this the points are sorted with respect to increasing function values and the best 10% are kept, the rest is discarded. On the remaining points we run the local optimization for 10 times more steps then before. This is iterated until only a certain number of points remain, out of which the one with the lowest function value will be output as the approximation for the global minimum.

# Binding Efficiency Index (BEI)

The Kd-based ligand binding efficiency index, BEI, was calculated by:

– log_10_(Kd_NMR_[M]/(Mw[kD])

The MIC-based ligand efficiency index, MIC-based BEI, was accordingly defined and calculated by:

– log_10_(MIC[M]/(Mw[kD])

Kd-based and IC_50_-based BEIs are quality parameters for oral small molecule drugs, lead compounds and screening hits ^3^. The 50% consensus group of marketed oral drugs with passive uptake shows BEIs between 15 and 35 ^3^, and lead compounds with Kd values ranging from 100 µM to 10 nM show BEIs between 17 and 25 for drugable enzymes, such as protein kinases ^4^. Here we introduce the MIC-based BEI because MIC values are the pivotal benchmarking parameters for antibacterial compounds irrespective of whether they originate from protein-based or whole cell anti-bacterial discovery programmes.

The BEI parameter is conceptually very similar to previously published ligand efficiency (LE) parameters that normalize logarithmic affinity by non-hydrogen atom count rather than molecular weight ^5-7^.

# Experimental Section

**Antibacterial in vitro assay for *M. smegmatis*.** For MIC assays according to the Clinical and Laboratory Standards Institute *M. smegmatis* (strain DSM 43465) cells were grown in CAMHB medium at 35°C to an OD_625nm_ higher than 0.09 followed by a dilution in medium to reach OD_625nm_ of 0.08 – 1 (“0.5 McFarland Standard”). These cell suspensions were brought to approximately 1 * 10^6^ CFU/ml which corresponds to a 1:200 dilution. Resulting cell inoculi were diluted 1:1 with medium containing increasing concentrations of test compound as indicated in 96 well plates in duplicates (cell titer and purity of inoculum was checked in parallel by plating out on CAMHB-agar). After 24 – 48 h incubation MTT-solution (5 mg/ml thiaolyl blue tetrazoliumbromide) were added to stain wells with living cells and plates were photographed ^8^. Wells with the lowest concentration of compound that were free of the dark brown color of bacteria were considered MICs. Ampicillin was used as a positive control antibiotic.

***M. tuberculosis (Mtb)* and *MDR-Mtb* in vitro growth inhibition assays.** MIC values for pathogenic multiple drug resistant *Mycobacterium tuberculosis* (MDR-*Mtb*, patient isolated strain 2745/09, National Reference Center for Mycobacteria, 23845 Borstel, Germany) were determined by using the radiometric BACTEC 460 method in liquid medium, previously validated for several TB antibiotics ^9,10^. Isoniazid was used as the reference antibiotic, showing growth of *Mtb-H37* (strain ATCC 27294) at 0.01 µg/mL isoniazid and no growth of *Mtb-H37* at 0.1 µg/mL isoniazid. As expected MDR-*Mtb* grew in the presence of both 0.01 and 0.1 µg/mL isoniazid.

# Supporting References

1 Moller, M. A scaled conjugate gradient algorithm for fast supervised learning. *Neural Networks* **1993**, *6*, 525-533.

2 Mayne, D. Q.Meewella, C. C. A non-clustering multistart algorithm for global optimization. *Lecture Notes in Control and Information Sciences* **1988**, *111*, 334-345.

3 Abad-Zapatero, C.Metz, J. T. Ligand efficiency indices as guideposts for drug discovery. *Drug Discov Today* **2005**, *10*, 464-469.

4 Hajduk, P. J. Fragment-based drug design: how big is too big? *J Med Chem* **2006**, *49*, 6972-6976.

5 Kuntz, I. D.; Chen, K.; Sharp, K. A.Kollman, P. A. The maximal affinity of ligands. *Proc Natl Acad Sci U S A* **1999**, *96*, 9997-10002.

6 Hopkins, A. L.; Groom, C. R.Alex, A. Ligand efficiency: a useful metric for lead selection. *Drug Discov Today* **2004**, *9*, 430-431.

7 Rees, D. C.; Congreve, M.; Murray, C. W.Carr, R. Fragment-based lead discovery. *Nat Rev Drug Discov* **2004**, *3*, 660-672.

8 Yajko, D. M.; Madej, J. J.; Lancaster, M. V.; Sanders, C. A.; Cawthon, V. L.; Gee, B.; Babst, A.Hadley, W. K. Colorimetric method for determining MICs of antimicrobial agents for Mycobacterium tuberculosis. *J Clin Microbiol* **1995**, *33*, 2324-2327.

9 Pfyffer, G. E. *et al.* Multicenter laboratory validation of susceptibility testing of Mycobacterium tuberculosis against classical second-line and newer antimicrobial drugs by using the radiometric BACTEC 460 technique and the proportion method with solid media. *J Clin Microbiol* **1999**, *37*, 3179-3186.

10 Heifets, L. B. in *Drug Susceptibility in the Chemotherapy of Mycobacterial Infections*, CRC Press: Boca Raton, 1991.
